# Cognitive and autonomic dysfunction in individuals with migraine: are they modifiable?

**DOI:** 10.1055/s-0045-1809419

**Published:** 2025-06-17

**Authors:** Raffaele Ornello

**Affiliations:** 1 University of L'Aquila, Department of Biotechnological and Applied Clinical Sciences, L'Aquila, Italy.

Migraine research has always been focused on finding biomarkers, i.e., findings that can distinguish individuals with migraine from those without migraine. However, the search for those biomarkers has never succeeded to date. While the structure of brains of the individuals with migraine does not differ from that of individuals without migraine, their functioning might be different. Clinical studies point toward an altered autonomic nervous system function in individuals with migraine compared with those without migraine, which suggests that clinical tests of the autonomic nervous system function might be useful when examining individuals with migraine. 1 The study of cortical functions in individuals with migraine compared with those without is challenging and has led to controversial results, 2 and it is currently debated whether individuals with migraine have a higher risk for dementia compared with those without migraine. 3

Keeping those challenges in mind, the authors of a cross-sectional study evaluated the cognitive and autonomic responses of individuals with migraine as compared with healthy controls via a device to detect brain activity and one to detect heart rate variability. While the performance in cognitive tests was similar between the two groups, individuals with migraine showed decreased brain activity and heart rate variability compared with the increased values found in controls without migraine. 4 Those findings can be interpreted in several ways. It could be argued that the brain of individuals with migraine has decreased activation compared with that of individuals without migraine after cognitive tasks. However, this conclusion does not fit with the similar cognitive abilities of individuals with and without migraine. It is more likely that the brains of individuals with migraines are more active than those of individuals without migraines at rest, therefore leading to reduced further activation with specific tasks. Research suggests that the brain of individuals with migraine is hyper-responsive to external stimuli. 5 Moreover, imaging evidence suggests an alteration of brain connectivity with an increase in connectivity of areas dedicated to pain perception. 6

As more and more studies are showing alterations in cortical and autonomic activity between individuals with migraine and those without, further evidence should be created on the possibility of modulating that activity with treatments. There is evidence showing that a substantial response to migraine preventive treatments, such as monoclonal antibodies targeting the calcitonin gene-related peptide, can reverse the hyper-responsiveness of the brain to external stimuli, leading to migraine-related autonomic activation. 7 8 It could be interesting to assess whether effective migraine prevention can lead to brain and autonomic activation levels comparable to those of individuals without migraine. “Normalization” of brain activity with effective treatments is an ambitious aim that can be obtained with migraine-specific preventive treatments, which showed high efficacy and excellent tolerability, allowing long-term treatments. 9

In conclusion, the recently published article is an invitation to pursue an objective study of brain activation and autonomic nervous system activity in individuals with migraine, as they could offer novel biomarkers for migraine diagnosis and management.
